# Minimal cut sets in metabolic networks: from conceptual foundations to applications in metabolic engineering and biomedicine

**DOI:** 10.1093/bib/bbaf188

**Published:** 2025-04-23

**Authors:** Steffen Klamt, Jürgen Zanghellini, Axel von Kamp

**Affiliations:** Analysis and Redesign of Biological Networks, Max Planck Institute for Dynamics of Complex Technical Systems, Sandtorstrasse 1, 39106 Magdeburg, Germany; Department of Analytical Chemistry, Faculty of Chemistry, University of Vienna, Sensengasse 8/15,1090 Vienna, Austria; Analysis and Redesign of Biological Networks, Max Planck Institute for Dynamics of Complex Technical Systems, Sandtorstrasse 1, 39106 Magdeburg, Germany

**Keywords:** metabolic networks, constraint-based modeling, computational strain design, metabolic engineering, robustness and fragility, mixed-integer linear programming

## Abstract

Minimal cut sets (MCSs) have emerged as an important branch of constraint-based metabolic modeling, offering a versatile framework for analyzing and engineering metabolic networks. Over the past two decades, MCSs have evolved from a theoretical concept into a powerful tool for identifying tailored metabolic intervention strategies and studying robustness and failure modes of metabolic networks. Successful (experimental) applications range from designing highly efficient microbial cell factories to targeting cancer cell metabolism. This review highlights key conceptual and algorithmic advancements that have transformed MCSs into a flexible methodology applicable to metabolic models of any size. It also provides a comprehensive overview of their applications and concludes with a perspective on future research directions. The review aims to equip both newcomers and experts with the knowledge needed to effectively leverage MCSs for metabolic network analysis and design, therapeutic targeting, and beyond.

## Introduction

Mathematical modeling techniques are nowadays routinely used to analyze properties and capabilities of metabolic networks and to determine intervention strategies that maximize the production of certain metabolites or block undesired metabolic functions. One key methodology for the computer-aided analysis of metabolic networks is constraint-based modeling [1, 2]. Founded on basic knowledge of the stoichiometric structure of the metabolic network and some linear equality or inequality constraints on metabolic fluxes, constraint-based modeling can be used to investigate an astonishing variety of important properties of metabolic networks and to make relevant predictions. First methodological advances in the 1990s led to the development of a phylogeny of techniques and methods firmly grounded on principles of constraint-based modeling [3]. One frequently used approach is flux balance analysis (FBA), which addresses the optimization of a certain metabolic objective, such as the maximization of growth rate [4]. Another class of methods is based on elementary (flux) modes, which facilitates the analysis of the smallest functional units (non-decomposable metabolic pathways) of metabolic networks [5, 6].

One branch of constraint-based modeling centers around the concept of minimal cut sets (MCSs). A first theoretical framework for MCSs in metabolic networks was presented in 2004 [7] with the goal to identify combinations of reaction deletions that block a certain metabolic function. Over the last 20 years, a number of follow-up works has then been published on theoretical properties and generalized concepts of MCSs as well as on algorithmic approaches for their computation. While applications focused initially on the analysis of properties of metabolic networks such as their structural robustness, the focus shifted more and more toward the determination of targeted intervention strategies in metabolic networks with successful case studies in metabolic engineering of microbial cell factories [8–11] or as a basis to identify therapeutic targets for disease states in human metabolism [12]. On the algorithmic side, while calculation of MCSs required initially the enumeration of elementary modes (EMs) in a preprocessing step (which is usually not possible in genome-scale metabolic networks), a major breakthrough was the development of an algorithm to compute the smallest MCSs in genome-scale models independently of the EMs [13]. With those and other algorithmic and conceptual advancements, the MCS approach developed into a very powerful and flexible framework for the computational design and targeted manipulation of metabolic networks. Its flexibility is also evident in the fact that many other optimization approaches for computing metabolic intervention strategies (e.g. OptKnock [14] for growth-coupled strain design or the determination of synthetic lethals [15]) can be represented as special MCS problems.

This article reviews (i) the evolution of the conceptual framework of MCSs and of algorithmic approaches for the calculation of MCSs (second section) and (ii) concrete applications of MCSs in metabolic engineering, biomedicine, and network analysis (third section). The following overview of theoretical and algorithmic developments aims to be as complete as possible while keeping mathematical details at a basic level and guiding novice as well as experienced readers interested in more theoretical insights to the relevant original publications.

## Evolution of conceptual definitions and enumeration algorithms for MCSs

### Basic definitions and terminology

This subsection introduces some basic terminology and concepts of stoichiometric and constraint-based modeling (for a more thorough introduction see [1, 2]).

We consider a metabolic network with $m$ metabolites and $n$ reactions represented by a *stoichiometric matrix*  $\mathbf{N}\in{\mathbb{R}}^{m\times n}$. In this matrix, the rows correspond to the metabolites and the columns to the reactions and ${\mathrm{N}}_{i,j}$ is the stoichiometric coefficient of metabolite $i$ in reaction $j$. With $\mathbf{r}\in{\mathbb{R}}^n$, we denote the vector of reaction rates (or fluxes). In constraint-based metabolic modeling, the focus is on the analysis of *steady-state flux distributions* where production and consumption of each (internal) metabolite are balanced, i.e. each metabolite has a constant concentration. In this case, the following equation holds:


(1) \begin{equation*} \mathbf{Nr}=\mathbf{0}. \end{equation*}

For irreversible reactions, we can demand that their rate cannot become negative (cannot run in backward direction):


(2) \begin{equation*} {\displaystyle \begin{array}{c}{r}_i\ge0, \; \; \forall i \in Irrev.\end{array}} \end{equation*}

Here, $Irrev$ is the set of indices of irreversible reactions. A more general variant of (2) is to specify for each reaction a lower and an upper bound for the reaction rate:


(3) \begin{equation*} {\displaystyle \begin{array}{c}{lb}_i\le{r}_i\le{ub}_i.\end{array}} \end{equation*}

One may also consider further linear inequalities of the form


(4) \begin{equation*} {\displaystyle \begin{array}{c}\mathbf{Ar}\le \mathbf{b},\end{array}} \end{equation*}

with a suitable matrix $\mathbf{A}\in{\mathbb{R}}^{q\times n}$ and vector $\mathbf{b}\in{\mathbb{R}}^{q\times 1}$, e.g. to express enzyme allocation constraints [16, 17].

The set of all flux vectors $\mathbf{r}$ fulfilling (1) and (2) span the so-called *flux cone*, while taking additionally constraints of type (3) or (4) into account leads to a geometric object called the *flux polyhedron* [6].

An important concept in the analysis of metabolic networks is that of *elementary (flux) modes* (EMs) [5, 6]. Every EM $\mathbf{e}$ is a particular flux vector of the flux cone, i.e. it obeys (1) and (2), and it fulfills the additional property of non-decomposability: there is no flux vector $\mathbf{r} \ne \mathbf{0}$ obeying (1) and (2) and whose support, $\mathit{\operatorname{supp}}\left(\mathbf{r}\right)$ is a proper (i.e. strict) subset of the support of $\mathbf{e}$. The support of a flux vector $\mathbf{r}$ is the set of the indices of the active reactions: $\mathit{\operatorname{supp}}\left(\mathbf{r}\right)=\left\{i\right|{r}_i\ne 0\}$. An EM with its support represents a minimal (irreducible) functional unit of a metabolic network: there is no proper subset of the active reactions of an EM that can maintain a non-zero steady-state flux in the network. Another important property is that any feasible steady-state flux vector $\mathbf{r}$ obeying (1) and (2) can be generated by a conic (non-negative) linear combination of all EMs:


(5) \begin{equation*} {\displaystyle \begin{array}{c}\mathbf{r}=\sum{\alpha}_i {\mathbf{e}}_{\boldsymbol{i}},\quad{\alpha}_i \ge 0.\end{array}} \end{equation*}

### A first concept and algorithm: MCSs blocking the operation of target reactions

The conceptual framework of MCSs has never been static and evolved in a very dynamic manner extending and generalizing previous definitions and developments. A first definition of (minimal) cut sets in metabolic networks was given in the seminal work [7] and relates to a (user-)selected *target (or objective) reaction* whose operation is to be blocked by the MCSs: a set of reactions is called a *cut set* (CS), if, after the removal of these reactions, all remaining feasible steady-state flux distributions have a zero flux for the target reaction. A CS is a *minimal cut set* (MCS) if no proper subset of it is a CS.

**Figure 1:**
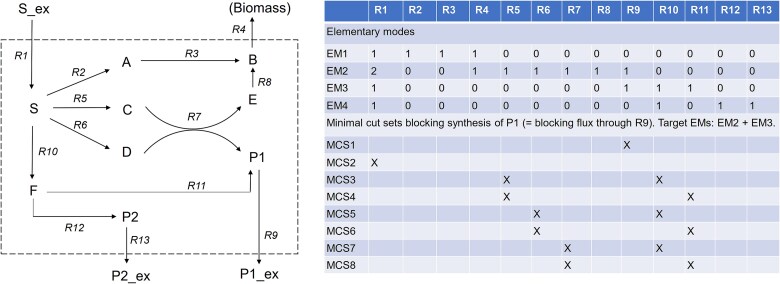
Example network with its EMs and with all MCSs blocking synthesis of P1 (target reaction: R9).

We consider the example network in Fig. 1 and assume that the target reaction is R9; hence, we aim to block synthesis of P1. This gives rise to eight MCSs, which, together with the four EMs of the network, are listed in the table in Fig. 1. Clearly, the MCS {R9} is trivial, but in realistic applications, it might not be a relevant intervention since the excretion (or diffusion) of metabolites can often not be prevented directly, except if a transporter is involved. It should also be noted that the minimality property of an MCS holds with respect to exclusion and not to its cardinality (the total number of reactions in the MCS). For this reason, in the example in Fig. 1, the six MCSs with two reactions are all minimal although two MCSs contain only one reaction. For example, blocking substrate uptake (reaction R1) is sufficient to disable synthesis of P1 (MCS2). In contrast, MCS3 requires deletion of R5 and R10; knocking-out only one of these two reactions would keep an alternative pathway to P1 functional. It can also be noted that some reactions are not contained in any MCS because none of the two pathways (EM2, EM3) to product P1 involves these reactions and their deletion would thus be of no benefit.

Although the original work [7] also allowed the consideration of multiple target reactions (which then had to be blocked simultaneously by the MCSs), the original MCS definition was limited in the scope of possible intervention problems. Furthermore, the definition did not allow for the protection of (desired) flux distributions in the network. For the example given above, we still might want to block synthesis of P1, but with the requirement that synthesis of P2 or biomass component B remains feasible. The MCS {R1} blocking uptake of the substrate is then no relevant option and should not be delivered by the algorithm. Despite these limitations of the initial definition, the work [7] already envisioned many interesting applications of MCSs (see below).

It is important to note that the demanded minimality property in the above MCS definition, which distinguishes an MCS from a CS, is similar to the non-decomposability property of EMs: both EMs and MCSs are elementary (or minimal or irreducible) in the sense that they cannot be further reduced without violating their central property. While the dual role of EMs as functional units and MCSs as minimal failure or intervention modes in metabolic networks was not yet clearly formulated, it was apparent that there are functional relationships between EMs and MCS.

This became also clear by the algorithm proposed in [7] for the computation of MCSs. It is based on the EMs of the network, which thus needed to be determined in a previous step. From the entire set of EMs, all EMs where the target reaction is active are selected for the MCS calculation. This set is called target EMs and denoted by ${\boldsymbol{E}}^{\boldsymbol{t}}$. The key idea here was that every MCS $\boldsymbol{C}$ must disable (or hit) all target EMs, i.e. for every target EM ${\mathbf{e}}_{\boldsymbol{i}}\in{\boldsymbol{E}}^{\boldsymbol{t}}$, the MCS must contain at least one reaction that is active in the target EM. Hence, for every MCS $\boldsymbol{C}$ it holds that


(6) \begin{equation*} {\displaystyle \begin{array}{c}\boldsymbol{C}\cap \mathit{\operatorname{supp}}\left({\mathbf{e}}_{\boldsymbol{i}}\right)\ne \varnothing, \; \; \forall{\mathbf{e}}_{\boldsymbol{i}} \in{\mathbf{E}}^{\boldsymbol{t}}\end{array}} \end{equation*}

and that no proper subset of $\boldsymbol{C}$ fulfills this property. With this definition, an MCS is a so-called *minimal hitting set* [18] of the set of target modes; however, this relationship was not yet clear at this time. The property of being a minimal hitting set means that deletion of the reactions from an MCS will block all target EMs and with this, due to eq. (6), also all steady-state flux vectors where the target reaction(s) are active. Based on this observation, the proposed iterative algorithm determines in the *n*-th iteration all MCSs of size *n* by combining each candidate set of size *n* − 1 with every single reaction and checking whether the obtained reaction set now hits all target EMs. If so (and if this reaction set is not a superset of previously found MCSs), then a new MCS has been found. If not, then the new set will be kept for the next iteration. Although this simple algorithm works correctly and was sufficient for example calculations in a core metabolic network of *Escherichia coli*, it is rather inefficient. In particular, it quickly runs out of memory since all subsets of size *n* have to be kept in the memory (up to the already found MCSs, which will not be further extended). What was not known to the authors at that time is a related but much better variant of their naive algorithmic scheme, namely, the Berge algorithm for computing minimal hitting sets (see below).

### A first generalization, minimal hitting sets and duality of MCSs and EMs

In the work [19], first generalizations of the original MCS definition were introduced. In particular, it was recognized that the target functionality (to be blocked by the MCSs) needs not be limited to the operation of a certain target reaction; instead, it can be any set of steady-state flux vectors that fulfill a given set of criteria. This extended definition allows, for example, to consider MCSs that (1) block all (non-zero) flux vectors that have, for a selected target product of interest, a product-substrate yield below a certain threshold, or (2) to block all flux vectors that use a certain metabolite as an intermediate compound. As in the previous work, for the calculation of the MCSs, the set of target modes needed to be properly selected based on the criteria for the flux vectors to be blocked from which the MCSs can then be computed (here still with the algorithm introduced in [7]). For example, in Fig. 1, if we aim to block all flux vectors where the yield of product P1 (per substrate S taken up) is <0.7 mol/mol, then the set of target modes comprises EM1, EM2, and EM4.

Although it was mentioned for the first time that the MCSs are the minimal hitting sets of the set of target modes, the Berge algorithm was still not applied. However, gaining knowledge about the theory of minimal hitting sets, a well-known concept in hypergraph theory [18], it was realized that there is a dual relationship between the MCSs and the set of target modes: the MCSs are the minimal hitting sets of the target EMs and, vice versa, the target EMs are the minimal hitting sets of the set of MCSs. This can indeed be verified by the EMs and MCSs shown in Fig. 1 for the case that operation of reaction R9 is to be blocked: not only does each MCS “hit” all target EMs (EM2 and EM3) according to eq. (5), it also holds that each of the two target EMs hits (intersects with) all MCSs.

Extending on these findings, the work of Haus *et al*. [20] gave a more precise and thorough characterization of the relationships between EMs and MCSs and the related theory of minimal hitting sets (in hypergraph theory, the latter are also known as minimal transversals of hypergraphs [18]). In this context, it was shown that the *Berge algorithm* previously developed to compute minimal hitting sets [21] is applicable to compute the MCSs from a given set of target modes. The authors implemented a version of the Berge algorithm in MATLAB (which became and still is part of *CellNetAnalyzer* [22, 23]), which included several preprocessing steps and smaller modifications to improve the runtime behavior of the algorithm. They showed that the Berge algorithm outperformed the first MCS algorithm presented in [7] by roughly two orders of magnitudes regarding both runtime and memory demand [20]. The key difference of the Berge algorithm is that it iterates over the EMs in the set of target modes which only slowly increases the size and the number of preliminary cut sets compared to the original algorithm, which keeps all possible subsets of reactions (quickly increasing the number of preliminary MCSs). Concretely, in each iteration, each preliminary cut set is tested whether it hits the currently processed target mode. If not, then the respective preliminary cut set gives rise to a new set of preliminary cut sets where the former is combined separately with each of the active reactions of the processed EM. Hence, if there are *z* many active reactions in the current EM, then *z* new preliminary cut sets are generated. These new MCS candidates are only kept if they are support-minimal w.r.t. to the other preliminary cut sets and, optionally, if the number of reaction cuts does not exceed a user-defined threshold. Afterwards, the next target EM is processed. Implementation variants of the Berge algorithm, e.g. via binary linear programming [24, 25] or with binary bit pattern trees to speed up the minimality tests of the preliminary MCSs [26], have been proposed, which can be useful for certain applications.

As was already illustrated in [19], Haus *et al*. [20] also highlighted a well-known fact from hypergraph theory, namely, that the computation of minimal hitting sets and thus of MCSs from the target EMs is equivalent to an operation known as dualization of monotone Boolean functions. The interesting consequence is that this allows, in principle, the use of an algorithmic scheme proposed by Fredman and Khachiyan [27], where, in each iteration, either a new MCS or a new (target) EM is generated—without the need to know the target EMs (or the MCSs) before. Instead, an oracle is used in each iteration which uses a classical FBA optimization problem to check whether a given set of reaction cuts blocks the target functionality or not. Although this algorithm has a guaranteed theoretical performance that is surprisingly very favorable (close to polynomial in the output size [27]), an implementation of the algorithm presented in [20] performed rather slowly and despite some recent improvements [28] this algorithm was rarely used in concrete applications.

### Taking desired (protected) functionalities into account: constrained MCSs

One disadvantage of the initial definitions of MCSs was that they could not directly account for desired functionalities that should be protected when blocking the undesired target functionality. This conceptual shortcoming was eliminated with the generalization of MCSs to constrained MCSs (cMCSs) [29]. The definition of cMCSs is straightforward: a constrained cut set is a set of reaction knockouts that blocks steady-state flux vectors with a given undesired (target) functionality while some flux vectors with a desired functionality must still be feasible after removal of the reactions contained in the cut set. A cMCS is a constrained cut set that cannot be further reduced.

For illustration, we use again the network in Fig. 1. As before, we assume that synthesis of P1 is to be blocked, but now we also demand that synthesis of P2 should remain feasible. It is easy to see that of the former eight MCSs blocking synthesis of P1, only four are valid for the formulated cMCS problem: MCSs 1, 4, 6, and 8. As already indicated in this example, the set of cMCSs is always a subset of the MCSs where the desired functionality is not taken into account. Hence, in principle, it is always possible to first compute the MCSs for the target functionality and filtering afterwards the cMCSs that also fulfill the requirement of feasibility of the protected desired functionality. However, when computing cMCSs, it turns out that it is often more efficient when all preliminary MCS candidates that violate feasibility of the desired functionality are eliminated already during the iterative computation of the cMCSs to reduce the set of preliminary candidates. The work [29] also presented an extension of the Berge algorithm for computing cMCSs (implemented and available in *CellNetAnalyzer*). As input, it accepts, as before, a given set of target modes and now additionally a given set of desired or protected modes. The user can select whether the feasibility of at least one desired mode is tested during the modified Berge algorithm (suggested) or afterwards. It is also possible to specify a number of desired modes that must remain functional.

The different (and partially equivalent) terms and meanings related to target/objective and desired/protected reactions/functionalities/modes (including the related definitions of target and desired regions that will later be discussed) are summarized in Table 1.

**Table 1:** Terms (with synonyms) related to target and desired reactions/functionalities/modes/regions and their respective meanings

**Term**	**Synonym**	**Meaning**
Target reaction	Objective reaction	Reaction whose operation is to be blocked by the MCSs
Target functionality/phenotype/behavior	Undesired functionality/phenotype/behavior	A specific functionality/phenotype/behavior to be blocked by the MCSs. Blocking the operation of a target reaction is one special case of a target functionality
Target modes/EMs	Undesired modes/EMs	All EMs that exhibit the target functionality (to be blocked by the MCS)
Target region	Undesired region; set of target flux vectors	The subset of all steady-state (target) flux vectors that exhibit the target functionality (to be blocked by the MCSs). If the target EMs are known, then the target region is spanned by these EMs. Alternatively, the target region can be described by suitable inequalities (eq. (7))
Desired functionality/phenotype/behavior	Protected functionality/phenotype/behavior	A desired functionality/phenotype/behavior that should remain feasible when deleting the reactions of an MCS
Desired modes/EMs	Protected modes/EMs	All EMs that exhibit the desired functionality (of which at least one EM must remain feasible when deleting all reactions of an MCS)
Desired region	Protected region	The subset of all steady-state flux vectors that exhibit the desired functionality (of which at least one must remain feasible when deleting all reactions of an MCS). If the desired EMs are known, then the desired region is spanned by these EMs. Alternatively, the desired region can be described by suitable inequalities (eq. (8))

As was also shown in [29], extending the concept of MCSs to cMCSs significantly broadens the scope of applications. In particular, cMCSs can now directly be used for posing and solving various problems in computational strain design, e.g. to compute metabolic engineering strategies that couple growth with synthesis of a product of interest (see below). Since almost all subsequent works on MCSs made use of the generalized definition of constrained MCSs (and because the original MCSs problem can be seen as a special case of cMCSs), the acronym MCSs was later also used for cMCSs and so we will do in the remainder of this paper if not stated otherwise.

### Direct computation of MCSs without EMs

Even with the extension to constrained MCSs, there were still two major limitations with the MCSs. First, their computation always required the determination of EMs in a first step, which is, due to combinatorial explosion of EMs, still not possible in genome-scale networks. Second, MCSs were so far only defined for flux vectors of the flux cone, i.e. only the two constraints (1) and (2) could be considered (these two constraints are called homogenous constraints because there are zeros on the right-hand side of the (in)equalities (1) and (2)). However, inhomogeneous constraints expressed by inequalities (3) and (4) are often highly relevant in metabolic models. For example, flux bounds used to limit the substrate uptake rate to measured values or to specify a minimum ATP demand required for non–growth-associated maintenance (usually denoted as ATPM flux) could not be taken into account. Likewise, more general linear inequalities over the fluxes (4), e.g. to specify molecular crowding [30] or enzyme capacity [17] constraints, could not be included. Only later it was shown that the Berge algorithm can also be used for computing MCSs if inhomogeneous constraints are included in the metabolic base model [6]; however, in this case, elementary flux vectors instead of EMs must be used as input whose computation can even be more complicated in large networks.

A simple brute-force procedure to compute MCSs directly is to use classical FBA to test systematically all 1-, 2-, 3-, … combinations of reaction knockouts whether the targeted flux vectors are blocked and the desired functionality remains feasible However, such an approach becomes quickly prohibitive for MCSs with larger cardinalities. For example, to demonstrate the combinatorial complexity of MCS calculation in genome-scale models, in a network with 2000 reactions, there would be up to 2.65·10^14^ candidate reaction knockout sets containing five reactions. Even if a computer performs 1000 FBA simulations per second, testing this limited set of candidates with five knockout combinations would already take >8400 years. Therefore, this simple brute-force approach has so far only been used to compute synthetic lethals (corresponding to MCSs’ blocking growth; see below) up to size three or so [15]. The mentioned Joint-Generation Algorithm of Fredman and Khachiyan [27], adapted for calculation of MCSs in [20, 28], is able to compute MCSs from scratch without knowing the EMs; however, it generates the EMs as byproduct and performed very slowly.

A major step forward in this direction was the work of Ballerstein *et al*. [31], showing that MCSs can be computed as EMs in a so-called dual network. Since target modes are now not available anymore, a step to be made was to express the target flux vectors to be blocked by linear inequalities of type


(7) \begin{equation*} {\displaystyle \begin{array}{c}\mathbf{Tr}\le \mathbf{t}\end{array}} \end{equation*}

with a suitable matrix $\mathbf{T}$ and vector $\mathbf{t}$. For example, if the goal is to prevent synthesis of product P1 in the network in Fig. 1, we could demand that all flux vectors with ${r}_9\ge 1$ need to be blocked (note that the 1 could be exchanged with any positive number; the result would always be the same because blocking all flux vectors with ${r}_9\ge 1$ also blocks all flux vectors with ${r}_9\ge a$ for any $a>0$). To bring this inequality into the correct orientation of (7), it can equivalently be written as $-{r}_9\le -1$ and we then get $\mathbf{T}=\left(0\ 0\ 0\ 0\ 0\ 0\ 0\ 0\ {-1} \ 0\ 0\ 0\ 0\right)$ and $\mathbf{t}=\left(-1\right)$. The target flux vector specification of type (7) is very flexible and may accommodate arbitrary linear inequalities. We also say that (7) together with the base model constraints (1)–(4) defines a *target region* since it represents geometrically a polyhedron within the space of the feasible steady-state fluxes of the given metabolic network model (cf. Table 1). As already indicated in the previous sentence, inclusion of the inhomogeneous flux constraints (3) and (4) is here indeed allowed, addressing a longstanding quest for further generalization.

The key finding in [31] is that the computation of MCSs is related to so-called irreducible inconsistent subsystems of a system of linear (in)equalities, which can in turn be determined as minimal solutions of an alternative linear system [32]. The alternative (dual) system (or dual network) can be derived from the *Farkas lemma*, a well-known result from linear programming. It turns out that the dual network can be described by another “stoichiometric” matrix which contains an identity matrix plus all flux constraints (1)–(4) and the constraints for the target fluxes (7) in the transposed version (hence, in particular, it includes ${\mathbf{N}}^{\boldsymbol{T}}$ and ${\mathbf{T}}^{\boldsymbol{T}}$). Thus, the former metabolites (rows in $\mathbf{N}$) represent now the reactions (columns) in ${\mathbf{N}}^{\boldsymbol{T}}$ and the former reactions the metabolites (see also figure 2 in [31]). The intriguing result is that a particular subset of the EMs of this dual stoichiometric network delivers the MCSs of the original (primal) network we are looking for. It is important to note that the duality property, which is exploited here, is different from the duality stating that MCSs can be computed as minimal hitting sets from the EMs and vice versa.

Ballerstein *et al*. [31] proved the theoretical results and showed that the MCSs of a core model of *E. coli*’s central metabolism can indeed be determined in this way. However, it was also observed that, with regard to the runtime, the new dual approach did not perform better than the former approach computing first the EMs and then the minimal hitting sets of the respective target EMs. One reason is that the dual network is significantly larger than the primal network and that it contains many EMs that are not relevant for the MCSs of the primal system (the issue of the larg dual system will be further discussed below). Furthermore, even if it is faster, it is clear that a full enumeration of the EMs in the dual (yielding the MCSs of the primal) would still not be possible in genome-scale networks due to combinatorial explosion of MCSs and EMs in these large systems.

These challenges were tackled by the MCSEnumerator approach presented in [13], which focuses on the enumeration of the smallest MCSs. It is based on a previous result showing that the shortest EMs of a metabolic network with the fewest number of active reactions can be computed iteratively (yielding first the smallest EM, then the second smallest EM and so on) by a mixed-integer linear programming (MILP) approach, even in genome-scale networks [33]. By combining a modified and improved variant of this algorithm together with the approach of Ballerstein *et al*., the smallest MCSs could now be computed as shortest EMs of the dual network [13]. Fortunately, it is indeed the smallest MCSs one is typically interested in. Realistic application examples demonstrated that this algorithm is able to list thousands of the most efficient intervention strategies in genome-scale networks for various intervention problems (including synthetic lethals and growth-coupled strain designs; see below). In the first MCSEnumerator variant presented in [13], constrained MCSs could be computed by checking feasibility of the desired (protected) behaviors or flux vectors for each calculated MCS. In later publications [34, 35], feasibility of the desired fluxes was ensured by directly integrating the constraint


(8) \begin{equation*} {\displaystyle \begin{array}{c}\mathbf{Dr}\le \mathbf{d}\end{array}} \end{equation*}

in the respective MILP formulation. Analogous to the target flux vectors in (7), matrix $\mathbf{D}$ and vector $\mathbf{d}$ are chosen such that they properly describe the desired metabolic behaviors (e.g. in Fig. 1, one may demand that synthesis of product P2 remains feasible). In analogy to the target region related to (7), the inequality (8) in combination with the base constraints (1)–(4) define the *desired region* of the defined space of steady-state fluxes of the metabolic model (cf. Table 1).

A further advancement in the context of the dual calculation of MCSs was presented by Miraskarshahi *et al*. [36]. As was already mentioned above, the dual network used by Ballerstein *et al*. [31] is always larger than the original (primal) network. In [36], the authors suggested the use of a more compact dual network based on the nullspace of the stoichiometric matrix which reduces the dimension of the problem compared to the Farkas lemma–based dual network of Ballerstein *et al*. They named their approach “minimal coordinated support MCS” (abbreviated MCS^2^). Together with further results presented in [37], using this nullspace-based dual network representation for the MILP-based enumeration of shortest MCSs reduces the runtime in the great majority of the studied cases (on average by a factor of 2.5 [37]).

**Figure 2:**
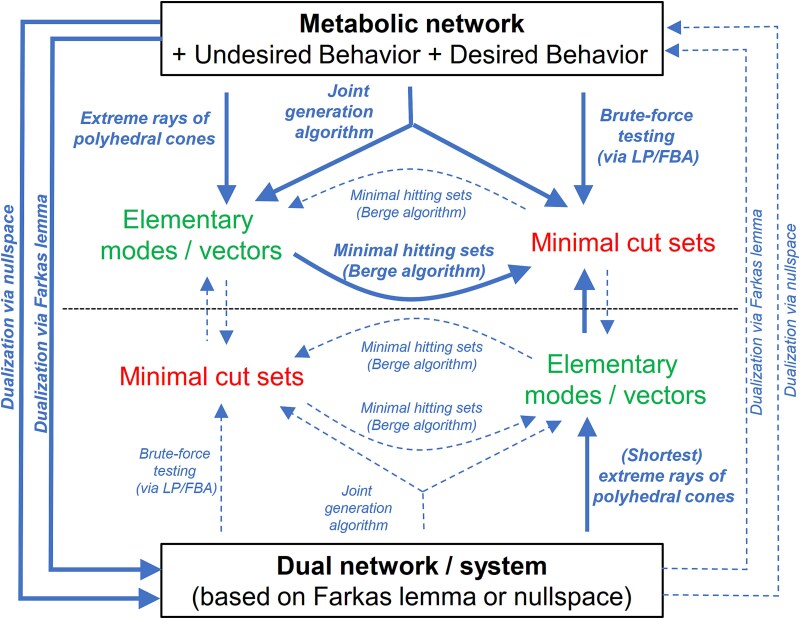
Relationships between MCSs, EMs, primal and dual network/system, and different ways of computing MCSs and EMs. Full (bold) lines represent routes that have been used for computations while dashed lines represent routes that are in principle possible but have not been utilized so far.

To conclude this section, the duality-based calculation of the shortest MCSs in a metabolic network, for arbitrary target and desired regions, was a cornerstone for the MCS framework because it facilitates the computation and analysis of (the most relevant) shortest MCSs in genome-scale networks. Since this method also frees the MCS approach from determining the EMs of the network in a preprocessing step, formerly used classifications of MCSs as “EM-based” (or “pathway-based”) design methods are not appropriate anymore. The relationships between the different MCSs calculation schemes and between MCSs and EMs are depicted in Fig. 2.

### MCSs targeting genes instead of reactions (genetic or gene MCSs)

For a long time, MCSs had been defined as sets of *reaction* deletions that solve a given intervention problem. However, in practice, genes (of the enzymes catalyzing the metabolic reactions) are the targets that can be knocked out in a cell rather than the reactions themselves. While it may in simple cases be relatively easy to translate an MCS of reactions to an MCS of genes, complex gene-protein-reaction (GPR) association rules are often prevalent in metabolic networks due to the existence of multifunctional (promiscuous) enzymes, isozymes, and of enzyme subunits that are required for different enzyme complexes. For example, knockout of the gene of a promiscuous enzyme may then block several reactions simultaneously. Luckily, these GPR rules are known for many organisms. In metabolic models, they are often formulated as Boolean equations that use AND and OR operators on genes to express which combinations of genes yield a functional enzyme catalyzing a given reaction.

The first approach to compute gene or genetic MCSs (gMCSs) was proposed by Machado *et al*. [38], in which the GPR associations are integrated in the stoichiometric model by adding the enzymes (or enzyme subunits) as pseudo-species to the network and connecting them with their associated reactions in the stoichiometric matrix. The enzymes or enzyme subunits are supplied by enzyme usage (pseudo-)reactions mimicking the expression of the associated genes. In this transformed model, a gene knockout can then be simulated by a knockout of the respective pseudo-reaction linked with that gene. Thus, gMCSs can then be computed as reaction knockouts of the respective enzyme usage reactions, while all other reactions are considered as non-targetable. By applying the dual (MCSEnumerator) approach to the transformed models with integrated GPR rules, the authors computed the smallest gMCSs for selected strain design problems and demonstrated that gMCSs may significantly differ from the original reaction MCSs (only 7% of the original reaction MCSs were feasible at the gene level [38]). Building upon the approach of Machado *et al*., the work [39] introduced a slightly different representation and several compression rules for GPR associations, which effectively sped up the computation of gMCSs in large-scale networks by one order of magnitude. Generally, while integrating GPR rules in the metabolic network enables the consideration of gene cuts, the target and desired behaviors are still specified in terms of reaction fluxes as the latter are more suitable to describe the respective phenotypes.

An alternative approach to compute gMCSs was presented by Planes and co-workers [12, 40]. Here, so-called G and F matrices are computed from the GPR rules in a preprocessing step. For each reaction, the G and F matrices contain one or several irreducible sets of gene knockouts that block this reaction (while other coincidentally blocked reactions are also marked). The G matrix is then integrated in the dual computation approach eventually delivering the gMCSs. This approach is limited in the sense that it cannot be used to compute constrained gMCSs where desired solutions are protected; however, especially with the latest gMCSpy implementation [41], it turns out that this approach is very efficient for computing synthetic lethalities (see also below).

### Regulatory MCSs, multiple target/desired regions, and gene/reaction additions

Another extension of the MCS framework has been proposed in [34] called *regulatory MCSs* (regMCSs). When calculating regMCSs, the classical reaction (or gene) deletions can be combined with up- and downregulation of certain reaction rates. The authors proposed a method by which those up- and downregulations of fluxes can be treated as virtual cuts allowing their direct integration in the existing dual MILP approach. However, allowing those regulatory interventions may vastly enlarge the search space in genome-scale networks and one therefore needs to preselect suitable candidates of fluxes to be considered for regulation. Applying this approach to identifying strain designs that lead to ethanol production in a genome-scale metabolic model of *E. coli* clearly showed that combining flux regulations and reaction deletions in regMCS may lead to interventions strategies that are significantly smaller than the conventional MCSs involving only knockouts [34].

**Figure 3:**
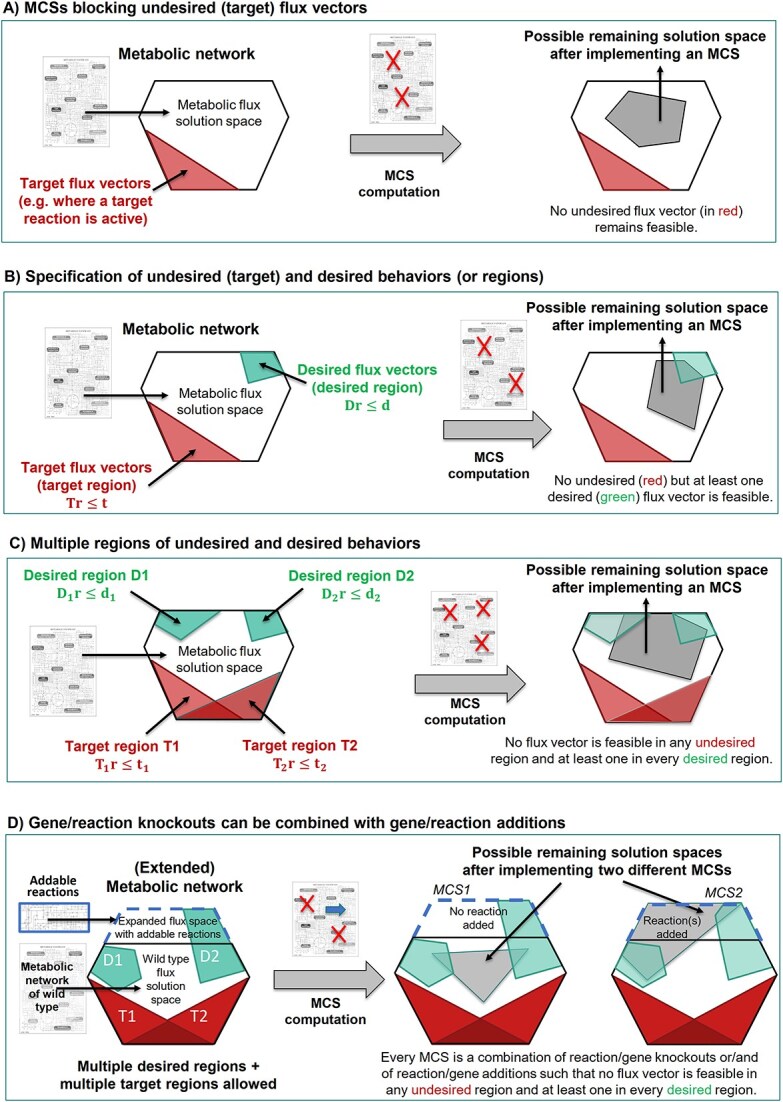
Evolution of major concepts of MCSs based on target (red) and desired (green) regions of the flux space. The regions can be defined either via linear inequalities (as indicated here) or via appropriate sets of elementary modes/elementary flux vectors, from which the MCSs can then be computed via the duality-based or the Berge algorithm, respectively.

Further generalizations of the MCS framework were presented in [39] broadening the scope of applications. In particular, it was shown how the definition of MCSs and the associated dual MILP algorithm can be extended from single target and desired regions to multiple target and desired regions. As demonstrated for the examples of designing strains for a two-stage process or for substrate co-feeding strategies, this enables a precise tailoring of the metabolic solution space with unlimited flexibility. Other generalizations include the use of individual cost factors for each intervention and the opportunity to combine reaction/gene deletions now also with reaction/gene additions (where an addition is counted as an intervention like a reaction or gene deletion). The evolution of major conceptual extensions and generalizations of MCSs are summarized in Fig. 3.

### Further algorithmic developments

An interesting approach to include metabolite concentrations into FBA and also MCS calculation has been developed in [42]. In its current form, it is possible to integrate (single substrate) irreversible Michaelis–Menten kinetics into the FBA which leads to a second-order cone program (SOCP). An SOCP can still be efficiently solved by an appropriate solver. It also has a dual formulation with which it is possible to calculate MCSs with reaction or metabolite knockouts that make a target region infeasible. Although metabolite knockouts may not be of primary interest in metabolic engineering, this new framework makes the inclusion of additional biological information into MCSs calculation possible leading to potentially more relevant results.

Some theoretical results on the complexity of calculations of EMs and MCSs were presented in [43]. It is shown that the enumeration of EMs and MCSs is computationally hard. Moreover, even the computation of the *minimum* cut set, which is the MCS with the smallest number of cuts (smallest cardinality), is NP-hard. Nevertheless, successful applications of the duality-based MCSEnumerator algorithm in genome-scale metabolic models have demonstrated that the determination of the smallest MCSs is feasible in realistic networks.

One important aspect in enumerating the smallest MCSs via the dual system is the handling of previously found solutions for which different approaches have been used. The simplest strategy is to iteratively solve the duality-based MILP, which minimizes the number of cuts to generate a new MCS and then adding an exclusion constraint that excludes supersets of the new solution from the solution space [13, 33]. Iterative computation of single MCSs has, in general, the disadvantage that a new MILP needs to be solved from scratch in each iteration and although modern MILP solvers have warm-start capabilities, these in practice only carry little information over to the next iteration. Therefore, a different strategy is to calculate all MCSs of a fixed size using the populate feature of modern MILP solvers which generates multiple/all solutions to a MILP. Starting this enumeration with cut sets of size one and successively increasing the cut set size and adding exclusion constraints for the newly found MCSs after each iteration generates MCSs with increasing size [13]. However, this method only works efficiently as long as the cut set size does not get too large (this number depends on the network size and the number of potential MCSs). Note that, in principle, it would be possible to use populate without fixing the cut set size (or only with setting an upper limit), but this would result in generating many non-minimal solutions which would be very inefficient. This problem can be circumvented with solution callbacks available in modern MILP solvers: When the solver finds a feasible solution, the callback is executed and within this callback an MCS is extracted from the solution (as above). Then, a lazy constraint is added to the problem to exclude supersets of the MCS from the solution space and the solver resumes its operation. This scheme has been implemented in the optlang_enumerator package [44], which is a part of the CNApy toolbox (see below) and can be used to efficiently generate MCSs of variable sizes up to the defined limit.

Generally, solving the duality MILP to optimality can take long, especially in genome-scale networks where even the smallest MCSs may have a large size. Alternatively, the MILP-solver can be configured to produce an integer-feasible solution which may be a non-minimal cut set. With a series of LPs it is then possible to extract an MCS from the initial solution. This is much faster than always solving the MILP to optimality, but usually does not produce MCSs with sizes in an increasing order.

Another issue of the duality-based approach is the incorporation and protection of the desired behaviors. Originally, the MILP was only used to calculate the unconstrained MCSs and the constrained MCSs accounting for the desired regions were then selected afterwards from this set via LPs [13]. However, this can be rather inefficient in situations where many unconstrained MCSs exist and only few of them fulfill the desired behavior(s) because exclusion constraints will also have to be added for non-constrained MCSs which greatly increases the MILP size. Therefore, the MILP was later adapted to include the desired behavior(s) [34, 35]. Also, the MILP can be formulated in various ways which can have a significant impact on the enumeration performance [37].

It is also possible to enumerate MCSs in large networks on a different basis than the duality-based MILPs. In one approach, a previously developed answer set programming (ASP)/LP framework for EMs computation was adapted for the calculation of MCSs [45]. The concrete application in this publication is the calculation of reaction cut sets that disable growth of a microbial community (see below). Given the flexibility of ASP, it is conceivable that multiple targets and desired regions as well as gene cut sets can be computed by extending the framework but at present this is not implemented.

Finally, another approach to (indirectly) calculate MCSs was suggested in [82]. The primary goal in this work was to calculate EMs using an alternation of LPs and integer linear programs (ILPs). The ILPs (intermittently) generate also MCSs that disable the set of EMs that is to be enumerated. By configuring this set in a manner that corresponds to a target region in the dual approach, it is possible to calculate the MCSs which disable this target region (and the EMs spanning the latter). Although it follows a different strategy, this approach shares some analogies to the Fredman–Khachiyan algorithm mentioned above, which also extracts EMs and MCS iteratively in some alternating order.

### Related notions of MCSs from other fields

There are several related notions of MCSs from other fields, many of which were already mentioned in the first paper [7]. For example, MCSs of fault trees studied in reliability and risk assessment of industrial systems [46] represent an irreducible combination of components or basic events that lead to a system failure. This can be translated into the notion of MCSs in metabolic networks: the basic events would be the deletion of single reactions and the system failure is the full blocking of the unwanted (target) flux vectors. However, since fault trees represent logical combinations of events, a direct application to metabolic networks, which are represented by a stoichiometric matrix, is not possible. Nevertheless, the computation of minimal hitting sets of the target modes to obtain the MCSs, which is related to the dualization of monotone Boolean function, shares some analogies with the computation of MCSs from fault trees.

Cut sets are also known from graph theory in quite different contexts and definitions. One definition is related to finding cuts that disconnect a graph. In this context, an *(edge) cut* is a set of edges that, if removed from a connected graph, will disconnect the graph in two or more components [47]. A *minimal edge cut(set)* is an edge cut such that if any edge is put back in the graph, the graph will be reconnected. This definition is refined if a dedicated source (*s*) and terminal (*t*) node is considered. An *s-t-cut(set)* is then a set of edges whose removal from a graph leaves no paths from the source *s* to the terminal *t*. A *minimal s-t-cutset* is a cutset where no proper subset of it is a cutset (note that in graph theory, cutset is often written as one word or as cut-set). A minimal s-t-cutset has again some analogies to MCSs in metabolic networks as it blocks any path or flow from *s* to *t*; however, as pointed out in [7, 48], at least a direct transfer of methods or algorithms from cutsets in graph theory is not possible due to the fact that metabolic networks are a special class of directed hypergraphs [49].

Another relationship between MCSs (and also EMs) exist to matroids [50, 51]. For example, if all reactions in a metabolic network are reversible, then the EMs are the so-called circuits of the matrix (or vector) matroid spanned by the stoichiometric matrix and the MCSs blocking all flux vectors and thus all EMs are then the circuits of a so-called dual matroid (also called co-circuits) [43].

Concepts of minimal intervention strategies have also been studied in other biological networks different from metabolism. For example, MCSs were computed in (interaction) graph models of signaling networks to interrupt signal flow along signaling paths or feedback loops [52]. In this particular case, the MCSs can indeed be computed with the algorithms described above, i.e. via the Berge algorithm if the signaling paths or/and feedback loops are known or via the dual (MILP-based) calculation, where the incidence matrix of the graph replaces the stoichiometric matrix [52]. Alternatively, the s-t-cutset algorithms for graphs (see above) could be used as well. A related concept in signaling or regulatory networks is *minimal intervention sets (MISs)* [52, 53]. However, MISs are defined in another formalism (Boolean or logical networks) where they represent minimal sets of permanent inactivations (nodes are permanently switched off) or activations (nodes are permanently switched on) of certain nodes to reach a desired behavior (intervention goal). Therefore, despite some analogies, the algorithm proposed for computing MISs [53] differs markedly from MCS-related algorithms.

## Applications of MCSs

### Strain design and metabolic engineering

#### Computing MCSs that couple cell growth and product synthesis

**Figure 4:**
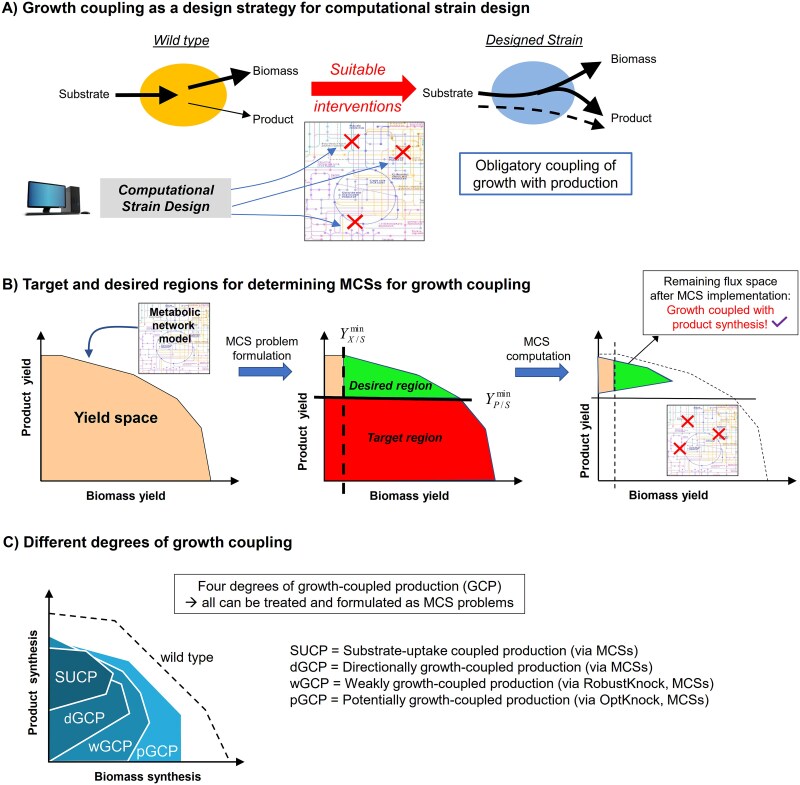
Use of MCSs for metabolic engineering and strain design. (A) Concept of growth-coupled strain design. (B) Formulating a MCS problem for growth-coupled strain designs. (C) Four different degrees of growth coupling and methods that can be used to compute associated strain designs.

MCSs represent intervention strategies in a metabolic network that block undesired phenotypes while maintaining desired metabolic behaviors. It is therefore not surprising that metabolic engineering and strain design is one major application field of MCSs, where the goal is to genetically modify the metabolism of microbial cell factories for the efficient fermentation-based production of chemicals. Various concepts and algorithms have been developed over the last 20 years for computational strain design based on stoichiometric and constraint-based models, with OptKnock [14], RobustKnock, [54], and minimal metabolic functionality [59] as one of the first published approaches (for a comprehensive review of such methods, see [64, 80, 81]). Most stoichiometric strain design methods are based on the principle of growth-coupled production. This means that, due to the imposed stoichiometric constraints of the implemented intervention, the cell can only grow if it also produces the desired product (Fig. 4A). One example of such a stoichiometric coupling would be the knockout of reactions R2 and R12 in the network in Fig. 1, through which synthesis of the biomass component B is coupled to production of product P1.

Coupling growth with production creates a selective pressure for the organism to increase the synthesis of the compound while optimizing cellular fitness, a property that can be exploited in adaptive laboratory evolution [55]. This makes growth-coupled production a robust and efficient strategy for metabolic engineering. In fact, using MCSs as computational framework, it has been shown that, under aerobic conditions, almost all endogenous (small) metabolites can in principle be produced in a growth-coupled manner in industrially relevant production organisms such as *E. coli*, the yeast *Saccharomyces cerevisiae*, *Corynebacterium glutamicum*, the filamentous fungus *Aspergillus niger*, and the cyanobacterium *Synechocystis* sp. PCC 6803 [35].

MCSs provide a straightforward way to enforce growth-coupled production in strain design. Figure 4B illustrates how MCS problems can be formulated that lead to growth-coupled product synthesis. It is useful to compute, in a first step, the two-dimensional biomass–product yield space, which displays the respective maximal yield of biomass and of the product of interest and the trade-offs between these different objectives. Yield spaces, or, alternatively, so-called production envelopes projecting the flux space on growth and production rates, can be easily computed from a given constrained-based genome-scale model [56]. Next, a minimum product yield is defined. The set of all flux vectors with a lower product yield forms the target region to be suppressed by the MCSs. To ensure that growth remains feasible, a minimum biomass yield is demanded and all flux vectors that lie above this and the product yield threshold are considered as desired behaviors from which at least one solution must remain feasible when an MCSs is genetically implemented. This simple specification of target and desired region defines an MCS problem where implementation of a computed MCS will guarantee that growth is only feasible if some minimum amount of product is synthesized.

First applications of MCSs to compute growth-coupled strain designs relied on the EM-based algorithm where the sets of target and desired EMs where defined according to the given thresholds for the demanded minimal biomass and product yields [29, 57, 58]. This approach is similar to the EM-based of minimal metabolic functionality (MMF) that was used by Trinh *et al*. in several studies, e.g. for the growth-coupled production of ethanol from hexoses [59] or for the construction of modular cells [60]. However, as pointed out in [29], the MMF approach usually delivers only one (and not necessarily the smallest) knockout strategy, whereas an MCS-based approach allows systematic exploration of all possible knockout strategies and thus a rigorous proof for the smallest number of required interventions. Since EM-based calculations are limited to core metabolic models, soon after the development of the dual (EM-free) algorithm for the calculation of the shortest MCSs (see above), MCS-based growth-coupled strain designs were mainly calculated by this approach, especially in genome-scale models [8–11, 35, 61–63].

Already in the first article using MCSs for the calculation of growth-coupled strain designs [29], it was shown that the MCS framework can also be employed to compute growth-coupled strain designs delivered by other methods such as OptKnock [14] and RobustKnock [54]. At this point, it is important to notice that different degrees of growth coupling can be defined that have recently been characterized in a systematic manner [64]. Basically, four different coupling degrees can be distinguished, whose characteristic production envelopes have been illustrated in Fig. 4C. The weakest of all coupling degrees is potentially growth-coupled product synthesis where it is only demanded that the product can, in principle, be synthesized at the maximal growth rate, but this is not guaranteed. OptKnock [14] is built on this principle, which is often overly optimistic, because even when focusing on the maximum growth rate, it is not ensured that competing pathways might divert metabolic flux away from the product of interest. The second class is weakly growth-coupled designs, where product synthesis at maximal growth rate is guaranteed by disabling competing pathways. RobustKnock [54] is built on this principle. For instance, in anaerobic fermentation of *E. coli* on glucose, acetate is necessarily produced to reach the maximal growth rate. However, at lower growth rates, acetate production is not essential. Hence, for enforcing weakly growth-coupled production of acetate, RobustKnock would just demand to block oxygen uptake (i.e. to switch off oxygen supply). However, this does not guarantee acetate synthesis if the cell grows with non-optimal growth rates. Here, the third coupling degree, directionally growth-coupled design, demands a stricter production feature: product synthesis is stoichiometrically enforced at every non-zero growth rate. That is, whenever the cell grows, product synthesis occurs. Interestingly, although this is the most natural formulation of a growth-coupling strategy, this coupling degree has rarely been considered in concrete calculations so far (with one exception of a purely theoretical study [65]). Finally, the strongest coupling degree is substrate uptake-coupled production (sometimes also called strong (growth) coupling), which requires that a product is produced whenever the substrate is utilized, even at zero growth. In fact, the MCS problem formulation for growth-coupled strain designs described above and in Fig. 4B follows this principle. One may argue that the MCSs calculated in this way demand more than necessary. However, this coupling degree also ensures product formation if cells do not grow but still take up substrate, e.g. for synthesizing ATP for (non-growth associated) maintenance demands. In this sense, not only growth but also ATP synthesis would be coupled to product formation, which could be relevant for two-stage fermentations with separated growth and production phase [64, 66]. As supported by empirical results [64], it is plausible to expect that, in general, the degree of coupling serves as a proxy for the required minimal number of necessary interventions, which increases with the strength of coupling.

While the MCS framework has so far mainly been used to compute growth-coupled strain designs with the strongest coupling degree, it has been shown that both the EM-based as well as the duality-based MCS algorithms can be used to compute intervention strategies also for the other coupling degrees [29, 64]. The key is to properly select the target and desired EMs (for the Berge algorithm) or the target and desired regions (for the duality-based MILP approach), to enforce the desired coupling degree. For the duality-based calculation, a major challenge was the implicit integration of the optimal growth rate in the MCS problem formulation, which is needed for the potentially and weakly growth-coupled designs. However, this could eventually be achieved closing the gap between MCS-based and bilevel-based strain design approaches (like OptKnock and RobustKnock) and enabling now the computation and comparison of strain designs for all coupling degrees within a single framework [64].

#### Examples of MCSs-guided metabolic engineering

The flexibility of the MCS approach for calculating reaction or/and gene knockout strategies for rational metabolic engineering has been leveraged in a number of applications. First studies were mostly theoretical and investigated the potential of growth-coupled strain designs for different microbial hosts and target products, e.g. for the production of terpenoids with *E. coli* and *S. cerevisiae* [57], for the synthesis of biofuels with cyanobacteria [58], for engineering *Pseudomonas putida* to utilize azo dyes for the production of the polyketide actinorhodin [61], or for growth-coupled production of biofuels from lignocellulosic material with *Clostridium thermocellum* [63].

The first work that experimentally validated an *in silico* computed MCS by corresponding *in vivo* gene knockouts was presented by Harder *et al*. [8]. In this study, a metabolic model was used to compute MCSs for the growth-coupled (heterologous) production of itaconic acid by *E. coli.* From the 945 MCSs computed, one MCS was selected and the corresponding genes were iteratively knocked out leading to a strain that produced itaconic acid with an excellent yield of 0.77 mol/(mol glucose) and high titer (32 g/L) in minimal medium with glucose and small amounts of glutamic acid. At the time, this yield was the highest achieved for heterologous itaconic acid production and provided the first strong evidence that MCSs can effectively and successfully guide rational metabolic engineering.

Another impressive application and experimental validation of MCSs was presented by Banerjee *et al*. [10]. Using a genome-scale model of *P. putida* KT2440, they computed MCSs for the growth-coupled production of the non-native product indigoidine, a sustainable pigment, with this organism as production host. They used omics data to select from the 63 found MCSs the most promising engineering strategy requiring as many as 14 simultaneous reaction interventions. They managed to implement 14 corresponding gene knockdowns using multiplex-CRISPRi in a *P. putida* strain and achieved a high product yield of 0.33 g indigoidine/g glucose (~50% of the maximum theoretical yield) and a titer of 26 g/L, the highest indigoidine titer to date with glucose minimal medium.

The same group used a similar approach combining MCS-based growth-coupled strain design, omics data analysis, and this time also adaptive laboratory evolution to develop another *P. putida* strain that produces indigoidine with very high yield from *para*-coumarate, a lignin-derived aromatic that holds promises to serve as a sustainable feedstock [11]. In another application, they combined target predictions from MCSs and OptKnock-based solutions to build a *P. putida* strain that produced isoprenol, a precursor for sustainable aviation fuel, with a titer of 3.5 g/L in a fed-batch fermentation [9]. MCSs and other strain design techniques were also combined in the work presented in [62], where the goal was to improve the production of cyanide, the main leaching agent for gold recovery, by *Bacillus megaterium*.

Despite these examples of successful experimental validation of MCS-based genetic engineering targets, it is important to stress that applying multiple knockouts is not only experimentally challenging due to the increased stress in the mutants but also sets high standards for the quality and reliability of the underlying genome-scale metabolic models. Therefore, it remains essential to iteratively apply knockouts, characterize the resulting mutants, and update the used network model to the resulting metabolic phenotype if necessary.

As another challenge, the MCS-based computation of growth-coupled strain designs in genome-scale metabolic models leads often to hundreds or thousands of possible candidates of intervention strategies. The procedure presented in [67] helps to characterize and rank these candidates via different criteria including, e.g. the number of required genetic interventions, the (theoretically) guaranteed minimal product yield, the robustness of growth coupling, the thermodynamic driving force of the used pathway, and many others. It has also been speculated that selecting a knockout strategy that maintains high pathway diversification and redundancy enhances the robustness of the cell factories [68]. Another study investigated the effect of adding constraints (in that work enzyme allocation constraints) to a given metabolic model with respect to the MCSs that induce growth-coupled synthesis of certain target products [17]. For example, the authors found that the minimum cardinality of all MCSs (i.e. the minimum number of required interventions) may increase or decrease, depending on the chosen product. In other words, additional constraints may reduce the necessity to delete certain reactions but they may also increase the size of the MCSs because the smallest MCSs that were formerly valid become invalid, e.g. because they violate the feasibility of desired phenotypes under the added constraints. Similar results were found in [84], where the MCSs of a genome-scale model of *E. coli* were compared to a smaller subnetwork of the latter comprising only the major pathways of the central metabolism.

Further applications of MCSs in the context of metabolic engineering that go beyond growth-coupled designs include the computation of interventions that induce metabolic dependencies between microbial species to design synthetic microbial communities [69] and the development of the metabolic valve enumerator (MoVE), which can be used to identify genetic intervention strategies that decouple two desired phenotypes, e.g. growth and production in two-stage fermentation processes [70].

### Synthetic lethality and biomedical applications

MCSs can also serve as a powerful computational framework for identifying synthetic lethals (SLs). In contrast to essential genes, whose individual deletion would prohibit growth of a cell or organism, SLs are minimal combinations of two or more non-essential genes whose simultaneous knockout is lethal [15]. Constraint-based metabolic models can be used to determine “metabolic” SLs, i.e. knockouts of metabolic genes (encoding for metabolic enzymes) or metabolic reactions, that disable growth in the model. For example, Suthers *et al*. [15] systematically enumerated all essential reactions and then all SL pairs and triplets of metabolic reactions in an *E. coli* genome-scale model. The search for SLs can be naturally formulated as an MCS problem, as it aligns with the initial use of MCSs to block the operation of target reactions. Here, the target flux vectors to be blocked are all those with a growth rate above a given threshold (in the simplest case just all flux vectors with a growth rate above zero). Since there are no desired behaviors to maintain, this makes SL identification a simpler MCS problem compared to strain design.

Already in the first paper on MCSs [7], minimal combinations of growth-disabling reaction deletions have been determined in a core metabolic model of *E. coli*, although they have not been termed synthetic lethals. In [13], SLs with up to five reaction deletions could be fully enumerated for the first time in a genome-scale model by calculating the corresponding MCSs with the duality-based algorithm. The MCSs approach for SLs calculation was two orders of magnitude faster than the method previously used in [15].

Biomedical applications of MCS-based calculations of SLs have been presented by Planes and co-workers in a couple of publications [12, 40, 41, 71]. In contrast to the previous SL calculations, they directly integrated GPR rules in the metabolic model before calculating SLs as MCSs and, thus, obtained gMCSs representing the SLs at the gene level (regarding the different techniques for computing MCSs at the gene level see above). In the work [12], this gMCS approach was used in combination with a human genome-scale model (Recon2.v04) and gene expression data from cell lines of multiple myeloma, a hematological cancer, to identify suitable genetic targets in the metabolism of these cancer cells that would stop their growth. This analysis predicted that ribonucleotide reductase catalytic subunit M1 (RRM1) is an essential gene in three of the four studied multiple myeloma cell lines, which could afterwards be validated in experiments.

The computation of SLs as gMCSs is supported by the developed dedicated toolboxes gMCS [40] and gMCSpy [41], respectively (see also below). Recently, the group also released gmcstool, a freely accessible online tool for the prediction and analysis of metabolic vulnerabilities of cancer cells based on the gMCS approach in combination with gene expression data [71]. In particular, it includes a database of synthetic lethals blocking certain metabolic tasks (including growth) in Human1, the most recent genome-scale metabolic model of human cells.

Another MCS-based computational study with a biomedical focus was presented in [45]. Here, SL strategies were determined to identify therapeutic targets blocking a microbial community of *Staphylococcus aureus* and *Pseudomonas aeruginosa*. These two bacterial pathogens often co-exist in chronic wounds and cystic fibrosis lungs promoting antimicrobial resistance. The authors determined a list of MCSs with promising enzyme targets for a consortium-level therapeutic application.

### Robustness analysis of metabolic networks and other applications

The fact that MCSs can be interpreted as potential failure modes can also be used to analyze and quantify the robustness of a given metabolic network with respect to selected metabolic functions. The most relevant metabolic function in microorganisms is growth. In this case, the entirety of MCSs representing the synthetic lethals can be used to assess how robust biomass synthesis in the metabolic network is against random perturbations or failures. For example, existence of many MCSs comprising only few targets, in the extreme case of MCSs with a single (essential) gene or reaction, will indicate a high fragility and thus low robustness.

**Table 2:** Features of various MCS computation and analysis tools (as of January 2025)

	**CellNetAnalyzer**	**CoBAMP**	**gMCS/gMCSpy**	**aspefm**	**CNApy (with opt-lang_enumerator)**	**Strain design (SD)**
Reference	[22,33]	[78]	[40]/[41]	[45]	[44, 75]	[74]
Platform	MATLAB	Python	MATLAB/Python		Python	Python
EM-based MCS calculation (hitting set algorithm)	✓	✗	✗	✗	✗	✗
Duality-based MILP	✓	✓	✓	(✓)	✓ (optionally via SD)	✓
Gene-based MCSs	✓	✓	✓	✗	✓ (via SD)	✓
Desired behaviors (cMCSs)	✓	✗	✗	✗	✓ (optionally via SD)	✓
Reaction/gene additions	✓	✗	✗	✗	✓ (optionally via SD)	✓
Multiple target/desired regions	✓	✗	✗	✗	✓ (optionally via SD)	✓
Variable costs for interventions	✓	✗	✗	✗	✓ (optionally via SD)	✓
GUI	✓	✗	✗	✗	✓	Via CNApy
Graphical display of MCSs	✓	✗	✗	✗	✓	Via CNApy
Tools for analysis of MCSs	✓	✗	✗	✗	✓	Via CNApy/Jupyter Notebook

A first robustness analysis of a metabolic network with respect to growth was already presented in [7]. For example, from the size distribution of the MCSs it was concluded that growth of *E. coli* with glucose as substrate is structurally more robust than with acetate as substrate. A first MCS-based quantitative measure of the metabolic network robustness or fragility was also proposed, called fragility coefficient. The latter takes the average size of the MCSs into account and reaches its highest value of one if all reactions or genes are essential in the network. However, this definition did not support a rigorous probabilistic view of robustness.

This limitation was overcome by concepts presented in [72]. In analogy to reliability theory, the authors exactly quantify the probability of (network) failure (PoF) for a given network function and prove that this measure can be computed from the corresponding MCSs for that function. They also show that the PoF can be reliably estimated also in genome-scale metabolic networks from the MCSs with the lowest cardinalities computed via the duality-based algorithm. These concepts were then used to compare the structural robustness of representatives of *Enterobacteriaceae* (e.g. *E. coli*) versus *Blattibacteriaceae* (e.g. the endosymbiont *Blattabacterium cuenoti* Bge) revealing a dramatically lower structural robustness of the highly specialized endosymbionts. It is believed that this reduced robustness was shaped by massive gene losses and is compensated by the fact that these organisms live in relatively constant environments with a rich supply of nutrients. A more comprehensive comparison of metabolic robustness was presented in [73], where the PoF measure was calculated and analyzed for 489 metabolic networks.

## Available tools for computation and analysis of MCSs

Several freely available software packages support the calculation and/or analysis of MCSs in metabolic network models, which are summarized in Table 2. All tools accept metabolic models in SBML format. Most of the discussed MCSs computation variants [13, 19, 20, 29, 34, 35, 39], including different EM-based and duality-based approaches, have originally been implemented in *CellNetAnalyzer* (CNA) [22, 23], many of which are still available in this toolbox. StrainDesign is a recently published toolbox with many advanced and unique features for MCSs computation [74]. In particular, StrainDesign is the only tool that supports the calculation of OptKnock-like or RobustKnock-like strain designs within the MCS framework. StrainDesign is written in Python and can be used via a GUI frontend in CNApy [75]. CNApy also contains its own MILP-based routine for calculation of MCSs via the optlang framework [76] (this package is called optlang_enumerator and may also be used autonomously [44]). With the exception of CNA and gMCS, which are MATLAB-based toolboxes (gMCS as package of the larger COBRA toolbox [77]), all other tools are implemented in Python. All tools rely on external MILP solvers or, in the case of aspefm [45], an external ASP solver.

## Discussion and conclusion

Since the introduction of metabolic MCSs two decades ago, they have driven major theoretical and algorithmic advancements, establishing MCSs as a key method for constraint-based metabolic modeling. These developments have deepened our understanding of theoretical properties of metabolic networks and enabled successful real-world applications. On the theoretical side, the key breakthrough was the characterization of the dual relationship between EMs and MCSs, which laid the foundation for the development of efficient algorithms for the calculation of MCSs also in large metabolic networks. While an exhaustive enumeration of MCS in genome-scale models remains infeasible due to combinatorial explosion, the most relevant (i.e. smallest) MCSs can now be computed in very large networks. On the application side, MCSs offer a powerful and flexible framework for identifying intervention strategies and failure modes in metabolic networks and most impactful applications have been their use for designing superior microbial cell factories and targeting cancer metabolism.

Apart from the main application fields reviewed herein, we believe that there are still many other potential applications of MCSs that have not been recognized yet. One example is the removal of erroneous energy-generating cycles of reconstructed metabolic networks as discussed in [79]. In the cited work, a variant of the GLOBALFIT algorithm was used to identify minimal sets of model changes that eliminate these cycles while maintaining required basic functionalities (such as growth). The used algorithm involves bilevel optimization techniques that often lead to long runtimes. It appears straightforward to recast this task as an MCS problem by selecting appropriate target and desired regions, which can then be solved with the optimized duality algorithm. Here, MCSs could thus also function as a tool for model building and curation. Another potential application of MCSs not used so far is to employ it as a diagnosis tool. For example, if the metabolism in an individuum or patient shows an abnormal or pathological phenotype, where one (or several) essential compound(s) cannot be synthesized or only in small amounts, then the calculation of MCSs that block the production of these metabolites would reveal all potential failure modes that could have caused the observed phenotype (e.g. combinations of mutations related to genes of metabolic enzymes). It will be interesting to see whether such an approach can once help diagnose complex metabolic disease states. Generally, the available software tools developed for the calculation and analysis of MCSs facilitates now widespread use of the MCS framework for various applications.

We also believe that there are still many things to be learned on the theoretical side. In particular, a more in-depth characterization of relationships of MCSs with similar notions from other disciplines, especially matroids, remains an interesting aspect for future work, as it may take us to concepts and algorithms that could be relevant for MCSs in metabolic networks as well (see e.g. [83]). Conceptual generalizations and broadening the scope of MCSs, also beyond metabolic networks in other fields of systems biology and network theory, are further promising directions of future research.

**Key Points**

- MCSs offer a versatile and powerful framework for identifying metabolic intervention strategies and studying robustness and failure modes of metabolic networks.
- This review summarizes key conceptual and algorithmic advancements of the last 20 years that have transformed MCSs into a flexible methodology applicable to metabolic models of large size.
- MCSs have been used and experimentally validated to guide the metabolic design of highly efficient microbial cell factories and for targeting cancer cell metabolism.

## References

[1] Bordbar A, Monk JM, King ZA. et al. Constraint-based models predict metabolic and associated cellular functions. *Nat Rev Genet* 2014;15:107–20. 10.1038/nrg3643 24430943

[2] Klamt S, Hädicke O, von Kamp A. Stoichiometric and constraint-based analysis of biochemical reaction networks. In: Benner P, Findeisen R, Flockerzi D. et al. (eds.), *Large-Scale Networks in Engineering and Life Sciences*, pp. 263–316. Heidelberg: Springer, 2014.

[3] Lewis NE, Nagarajan H, Palsson BO. Constraining the metabolic genotype–phenotype relationship using a phylogeny of in silico methods. *Nat Rev Microbiol* 2012;10:291–305. 10.1038/nrmicro2737 22367118 PMC3536058

[4] Orth JD, Thiele I, Palsson BO. What is flux balance analysis? *Nat Biotechnol* 2010;28:245–8. 10.1038/nbt.1614 20212490 PMC3108565

[5] Schuster S, Fell DA, Dandekar T. A general definition of metabolic pathways useful for systematic organization and analysis of complex metabolic networks. *Nat Biotechnol* 2000;18:326–32. 10.1038/73786 10700151

[6] Klamt S, Regensburger G, Gerstl MP. et al. From elementary flux modes to elementary flux vectors: metabolic pathway analysis with arbitrary linear flux constraints. *PLoS Comput Biol* 2017;13:e1005409. 10.1371/journal.pcbi.1005409 28406903 PMC5390976

[7] Klamt S, Gilles ED. Minimal cut sets in biochemical reaction networks. *Bioinformatics* 2004;20:226–34. 10.1093/bioinformatics/btg395 14734314

[8] Harder B-J, Bettenbrock K, Klamt S. Model-based metabolic engineering enables high yield itaconic acid production by *Escherichia coli*. *Metab Eng* 2016;38:29–37. 10.1016/j.ymben.2016.05.008 27269589

[9] Banerjee D, Yunus IS, Wang X. et al. Genome-scale and pathway engineering for the sustainable aviation fuel precursor isoprenol production in Pseudomonas putida. *Metab Eng* 2024;82:157–70. 10.1016/j.ymben.2024.02.004 38369052

[10] Banerjee D, Eng T, Lau AK. et al. Genome-scale metabolic rewiring improves titers rates and yields of the non-native product indigoidine at scale. *Nat Commun* 2020;11:5385. 10.1038/s41467-020-19171-4 33097726 PMC7584609

[11] Eng T, Banerjee D, Menasalvas J. et al. Maximizing microbial bioproduction from sustainable carbon sources using iterative systems engineering. *Cell Rep* 2023;42:113087. 10.1016/j.celrep.2023.113087 37665664

[12] Apaolaza I, José-Eneriz ES, Tobalina L. et al. An in-silico approach to predict and exploit synthetic lethality in cancer metabolism. *Nat Commun* 2017;8:459. 10.1038/s41467-017-00555-y 28878380 PMC5587678

[13] von Kamp A, Klamt S. Enumeration of smallest intervention strategies in genome-scale metabolic networks. *PLoS Comput Biol* 2014;10:e1003378. 10.1371/journal.pcbi.1003378 24391481 PMC3879096

[14] Burgard AP, Pharkya P, Maranas CD. Optknock: a bilevel programming framework for identifying gene knockout strategies for microbial strain optimization. *Biotechnol Bioeng* 2003;84:647–57. 10.1002/bit.10803 14595777

[15] Suthers PF, Zomorrodi A, Maranas CD. Genome-scale gene/reaction essentiality and synthetic lethality analysis. *Mol Syst Biol* 2009;5:301. 10.1038/msb.2009.56 19690570 PMC2736653

[16] Sanchez BJ, Zhang C, Nilsson A. et al. Improving the phenotype predictions of a yeast genome-scale metabolic model by incorporating enzymatic constraints. *Mol Syst Biol* 2017;13:935. 10.15252/msb.20167411 28779005 PMC5572397

[17] Bekiaris PS, Klamt S. Automatic construction of metabolic models with enzyme constraints. *BMC Bioinformatics* 2020;21:19. 10.1186/s12859-019-3329-9 31937255 PMC6961255

[18] Gainer-Dewar A, Vera-Licona P. The minimal hitting set generation problem: algorithms and computation. *SIAM J Discrete Math* 2017;31:63–100. 10.1137/15M1055024

[19] Klamt S . Generalized concept of minimal cut sets in biochemical networks. *Biosystems* 2006;83:233–47. 10.1016/j.biosystems.2005.04.009 16303240

[20] Haus UU, Klamt S, Stephen T. Computing knock-out strategies in metabolic networks. *J Comput Biol* 1008;15:259–68. 10.1089/cmb.2007.0229 18331197

[21] Berge C . Hypergraphs: Combinatorics of Finite Sets. Oxford: Elsevier, 1984.

[22] Klamt S, Saez-Rodriguez J, Gilles ED. Structural and functional analysis of cellular networks with CellNetAnalyzer. *BMC Syst Biol* 2007;1:2. 17408509 10.1186/1752-0509-1-2 PMC1847467

[23] von Kamp A, Thiele S, Hädicke O. et al. Use of *CellNetAnalyzer* in biotechnology and metabolic engineering. *J Biotechnol* 2017;261:221–8. 10.1016/j.jbiotec.2017.05.001 28499817

[24] Jungreuthmayer C, Zanghellini J. Designing optimal cell factories: integer programming couples elementary mode analysis with regulation. *BMC Syst Biol* 2012;6:103. 10.1186/1752-0509-6-103 22898474 PMC3560272

[25] Jungreuthmayer C, Nair G, Klamt S. et al. Comparison and improvement of algorithms for computing minimal cut sets. *BMC Bioinformatics* 2013;14:318. 10.1186/1471-2105-14-318 24191903 PMC3882775

[26] Jungreuthmayer C, Beurton-Aimar M, Zanghellini J. Fast computation of minimal cut sets in metabolic networks with a Berge algorithm that utilizes binary bit pattern trees. *IEEE/ACM Trans Comput Biol Bioinform* 2013;10:1329–33. 10.1109/tcbb.2013.116 24062540

[27] Fredman ML, Khachiyan L. On the complexity of dualization of monotone disjunctive normal forms. *J Algorithms* 1996;21:618–28. 10.1006/jagm.1996.0062

[28] Sedaghat N, Stephen T, Chindelevitch L. Speeding up the structural analysis of metabolic network models using the Fredman–Khachiyan algorithm B. *J Comput Biol* 2023;30:678–94. 10.1089/cmb.2022.0319 37327036

[29] Hädicke O, Klamt S. Computing complex metabolic intervention strategies using constrained minimal cut sets. *Metab Eng* 2011;13:204–13. 10.1016/j.ymben.2010.12.004 21147248

[30] Vazquez A, Beg QK, deMenezes MA. et al. Impact of the solvent capacity constraint on E. coli metabolism. *BMC Syst Biol* 2008;2:7. 10.1186/1752-0509-2-7 18215292 PMC2270259

[31] Ballerstein K, von Kamp A, Klamt S. et al. Minimal cut sets in a metabolic network are elementary modes in a dual network. *Bioinformatics* 2012;28:381–7. 10.1093/bioinformatics/btr674 22190691

[32] Parker M, Ryan J. Finding the minimum weight IIS cover of an infeasible system of linear inequalities. *Ann Math Artif Intell* 1996;17:107–26. 10.1007/BF02284626

[33] De Figueiredo LF, Podhorski A, Rubio A. et al. Computing the shortest elementary flux modes in genome-scale metabolic networks. *Bioinformatics* 2009;25:3158–65. 10.1093/bioinformatics/btp564 19793869

[34] Mahadevan R, von Kamp A, Klamt S. Genome-scale strain designs based on regulatory minimal cut sets. *Bioinformatics* 2015;31:2844–51. 10.1093/bioinformatics/btv217 25913205

[35] von Kamp A, Klamt S. Growth-coupled overproduction is feasible for almost all metabolites in five major production organisms. *Nat Commun* 2017;8:15956. 10.1038/ncomms15956 28639622 PMC5489714

[36] Miraskarshahi R, Zabeti H, Stephen T. et al. MCS2: minimal coordinated supports for fast enumeration of minimal cut sets in metabolic networks. *Bioinformatics* 2019;35:i615–23. 10.1093/bioinformatics/btz393 31510702 PMC6612898

[37] Klamt S, Mahadevan R, von Kamp A. Speeding up the core algorithm for the dual calculation of minimal cut sets in large metabolic networks. *BMC Bioinformatics* 2020;21:510. 10.1186/s12859-020-03837-3 33167871 PMC7654042

[38] Machado D, Herrgård MJ, Rocha I. Stoichiometric representation of gene–protein–reaction associations leverages constraint-based analysis from reaction to gene-level phenotype prediction. *PLoS Comput Biol* 2016;12:e1005140. 10.1371/journal.pcbi.1005140 27711110 PMC5053500

[39] Schneider P, von Kamp A, Klamt S. An extended and generalized framework for the calculation of metabolic intervention strategies based on minimal cut sets. *PLoS Comput Biol* 2020;16:e1008110. 10.1371/journal.pcbi.1008110 32716928 PMC7410339

[40] Apaolaza I, Valcarcel LV, Planes FJ. gMCS: fast computation of genetic minimal cut sets in large networks. *Bioinformatics* 2018;35:535–7. 10.1093/bioinformatics/bty656 30052768

[41] Rodriguez-Flores CJ, Barrena N, Olaverri-Mendizabal D. et al. gMCSpy: efficient and accurate computation of genetic minimal cut sets in python. *Bioinformatics* 2024;40:btae318. 10.1093/bioinformatics/btae318 38748994 PMC11199197

[42] Taylor JA, Rapaport A, Dochain D. Convex representation of metabolic networks with Michaelis–Menten kinetics. *Bull Math Biol* 2024;86:65. 10.1007/s11538-024-01293-1 38671332 PMC11052807

[43] Acuna V, Chierichetti F, Lacroix V. et al. Modes and cuts in metabolic networks: complexity and algorithms. *Biosystems* 2009;95:51–60. 10.1016/j.biosystems.2008.06.015 18722501

[44] optlang_enumerator : https://github.com/cnapy-org/optlang_enumerator

[45] Mahout M, Carlson R, Simon L. et al. Logic programming-based minimal cut sets reveal consortium-level therapeutic targets for chronic wound infections. *Npj Syst Biol Appl* 2024;10:34. 10.1007/s11538-024-01293-1 38565568 PMC10987626

[46] Sinnamon R, Andrews J. New approaches to evaluating fault trees. *Reliab Eng Syst Saf* 1997;58:89–96. 10.1016/S0951-8320(96)00036-1

[47] Bollabas B . Modern Graph Theory. New York: Springer, 1998.

[48] Clark ST, Verwoerd WS. Minimal cut sets and the use of failure modes in metabolic networks. *Metabolites* 2012;2:567–95. 10.3390/metabo2030567 24957648 PMC3901212

[49] Klamt S, Haus UU, Theis FJ. Hypergraphs and cellular networks. *PLoS Comput Biol* 2019;5:e1000385. 10.1371/journal.pcbi.1000385 PMC2673028 19478865

[50] Oxley JG . Matroid Theory (Oxford Graduate Texts in Mathematics). Oxford: Oxford University Press, 2006.

[51] Welsh DJA . Matroid Theory. Dover: Courier Dover Publication, 2010.

[52] Klamt S, Saez-Rodriguez J, Lindquist JA. et al. A methodology for the structural and functional analysis of signaling and regulatory networks. *BMC Bioinformatics* 2006;7:56. 16464248 10.1186/1471-2105-7-56 PMC1458363

[53] Samaga R, von Kamp A, Klamt S. Computing combinatorial intervention strategies and failure modes in signaling networks. *J Comput Biol* 2010;17:39–53. 10.1089/cmb.2009.0121 20078396

[54] Tepper N, Shlomi T. Predicting metabolic engineering knockout strategies for chemical production: accounting for competing pathways. *Bioinformatics* 2010;26:536–43. 10.1093/bioinformatics/btp704 20031969

[55] Godara A . Kao KC adaptive laboratory evolution for growth coupled microbial production. *World J Microbiol Biotechnol* 2020;36:175. 10.1007/s11274-020-02946-8 33083911

[56] Klamt S, Müller S, Regensburger G. et al. A mathematical framework for yield (vs. rate) optimization in constraint-based modeling and applications in metabolic engineering. *Metab Eng* 2018;47:153–69. 10.1016/j.ymben.2018.02.001 29427605 PMC5992331

[57] Gruchattka E, Hädicke O, Klamt S. et al. In silico profiling of Escherichia coli and Saccharomyces cerevisiae as terpenoid factories. *Microb Cell Fact* 2013;12:84. 10.1186/1475-2859-12-84 24059635 PMC3852115

[58] Erdrich P, Steuer R, Knoop H. et al. Cyanobacterial biofuels: new insights and strain design strategies revealed by computational modeling. *Microb Cell Fact* 2014;13:128. 25323065 10.1186/s12934-014-0128-x PMC4180434

[59] Trinh CT, Unrean P, Srienc F. Minimal Escherichia coli cell for the most efficient production of ethanol from hexoses and pentoses. *Appl Environ Microbiol* 2008;74:3634–43. 10.1128/AEM.02708-07 18424547 PMC2446564

[60] Trinh CT, Liu Y, Conner DJ. Rational design of efficient modular cells. *Metab Eng* 2015;32:220–31. 10.1016/j.ymben.2015.10.005 26497627

[61] Nayyara P, Permana D, Ermawar RA. et al. Computational analysis into the potential of azo dyes as a feedstock for actinorhodin biosynthesis in pseudomonas putida. *PloS One* 2024;19:e0299128. 10.1371/journal.pone.0299128 38437212 PMC10911627

[62] Aminian-Dehkordi J, Mousavi SM, Marashi SA. et al. A systems-based approach for cyanide overproduction by Bacillus megaterium for gold bioleaching enhancement. *Front Bioeng Biotechnol* 2020;8:528. 10.3389/fbioe.2020.00528 32582661 PMC7283520

[63] Thompson RA, Dahal S, Garcia S. et al. Exploring complex cellular phenotypes and model-guided strain design with a novel genome-scale metabolic model of Clostridium thermocellum DSM 1313 implementing an adjustable cellulosome. *Biotechnol Biofuels* 2016;9:194. 27602057 10.1186/s13068-016-0607-x PMC5012057

[64] Schneider P, Radhakrishnan M, Klamt S. Systematizing the different notions of growth-coupled product synthesis and a single framework for computing corresponding strain designs. *Biotechnol J* 2021;16:e2100236. 10.1002/biot.202100236 34432943

[65] Tervo CJ, Reed JL. Expanding metabolic engineering algorithms using feasible space and shadow price constraint modules. *Metab Eng Commun* 2014;1:1–11. 10.1016/j.meteno.2014.06.001 25478320 PMC4249821

[66] Klamt S, Mahadevan R, Hädicke. When do two-stage processes outperform one-stage processes? *Biotechnol J* 2018;13:1700539. 10.1002/biot.201700539 29131522

[67] Schneider P, Klamt S. Characterizing and ranking computed metabolic engineering strategies. *Bioinformatics* 2019;35:3063–72. 10.1093/bioinformatics/bty1065 30649194 PMC6735923

[68] Yang L, Srinivasan S, Mahadevan R. et al. Characterizing metabolic pathway diversification in the context of perturbation size. *Metab Eng* 2014;28:114–22. 10.1016/j.ymben.2014.11.013 25542850

[69] Koch S, Kohrs F, Lahmann P. et al. RedCom: a strategy for reduced metabolic modeling of complex microbial communities and its application for analyzing experimental datasets from anaerobic digestion. *PLoS Comput Biol* 2019;15:e1006759. 10.1371/journal.pcbi.1006759 30707687 PMC6373973

[70] Venayak N, von Kamp A, Klamt S. et al. MoVE identifies metabolic valves to switch between phenotypic states. *Nat Commun* 2018;9:5332. 10.1038/s41467-018-07719-4 30552335 PMC6294006

[71] Valcárcel LV, José-Enériz ES, Ordoñez R. et al. An automated network-based tool to search for metabolic vulnerabilities in cancer. *Nat Commun* 2024;15:8685. 39394196 10.1038/s41467-024-52725-4 PMC11470099

[72] Gerstl MP, Klamt S, Jungreuthmayer C. et al. Exact quantification of cellular robustness in genome-scale metabolic networks. *Bioinformatics* 2016;32:730–7. 10.1093/bioinformatics/btv649 26543173 PMC4795620

[73] Libiseller-Egger J, Coltman BL, Gerstl MP. et al. Environmental flexibility does not explain metabolic robustness. *Npj Syst Biol Appl* 2020;6:39. 10.1038/s41540-020-00155-5 33247119 PMC7695710

[74] Schneider P, Bekiaris PS, von Kamp A. et al. StrainDesign: a comprehensive python package for computational design of metabolic networks. *Bioinformatics* 2022;38:4981–3. 10.1093/bioinformatics/btac632 36111857 PMC9620819

[75] Thiele S, von Kamp A, Bekiaris PS. et al. CNApy: a CellNetAnalyzer GUI in Python for analyzing and designing metabolic networks. *Bioinformatics* 2022;38:1467–9. 10.1093/bioinformatics/btab828 34878104 PMC8826044

[76] Jensen K, Cardoso JGR, Sonnenschein N. Optlang: an algebraic modeling language for mathematical optimization. *J Open Source Softw* 2017;2:139. 10.21105/joss.00139

[77] Heirendt L. et al. Creation and analysis of biochemical constraint-based models: the COBRA toolbox v3.0. *Nat Protoc* 2019;14:639–702. 10.1038/s41596-018-0098-2 30787451 PMC6635304

[78] Vieira V, Rocha M. CoBAMP: a Python framework for metabolic pathway analysis in constraint-based models. *Bioinformatics* 2019;35:5361–2. 10.1093/bioinformatics/btz598 31359031

[79] Fritzemeier CJ, Hartleb D, Szappanos B. et al. Erroneous energy-generating cycles in published genome scale metabolic networks: identification and removal. *PLoS Comput Biol* 2017;13:e1005494. 10.1371/journal.pcbi.1005494 28419089 PMC5413070

[80] Machado D, Herrgård MJ. Co-evolution of strain design methods based on flux balance and elementary mode analysis. *Metab Eng Commun* 2015;2:85–92. 10.1016/j.meteno.2015.04.001 34150512 PMC8193246

[81] Maia P, Rocha M, Rocha I. In silico constraint-based strain optimization methods: the quest for optimal cell factories. *Microbiol Mol Biol* 2016;80:45–67. 10.1128/MMBR.00014-15 PMC4711187 26609052

[82] Song HS, Goldberg N, Mahajan A. et al. Sequential computation of elementary modes and minimal cut sets in genome-scale metabolic networks using alternate integer linear programming. *Bioinformatics* 2017;33:2345–53. 10.1093/bioinformatics/btx171 28369193

[83] Röhl A, Riou T, Bockmayr A. Computing irreversible minimal cut sets in genome-scale metabolic networks via flux cone projection. *Bioinformatics* 2018;35:2618–25. 10.1093/bioinformatics/bty1027 30590390

[84] Hädicke O, Klamt S. EColiCore2: a reference model of the central metabolism of Escherichia coli and the relationships to its genome-scale parent model. *Sci Rep* 2017;7:39647. 10.1038/srep39647 28045126 PMC5206746

